# Heart rate variability as a predictor of successful catheter-guided pulmonary vein isolation for atrial fibrillation

**DOI:** 10.1007/s00059-023-05201-6

**Published:** 2023-08-17

**Authors:** M. Drexler, T. Blum, K. M. Heinroth, T. Hartkopf, A. Plehn, P. Schirdewahn, D. G. Sedding

**Affiliations:** 1 Klinik für Innere Medizin III—Kardiologie, Angiologie und Internistische Intensivmedizin, Universitätsklinikum Halle (Saale), Ernst-Grube-Str. 40, 06120 Halle (Saale), Germany; 2 Praxisklinik Salzatal, An der Lehmwand 2, 06198 Salzmünde, Germany; 3 Kardiologische Praxis Dr. Petra Schirdewahn, Schillerpl. 12, 06198 Salzmünde, Germany; 4 Merseburger Str. 96, 06110 Halle (Saale), Germany; 5 Martin-Luther-Universität Halle-Wittenberg (1043), Halle (Saale), Germany

**Keywords:** Atrial fibrillation recurrence, Holter monitoring, Catheter ablation, Cryoballoon, Radiofrequency ablation, Vorhofflimmern Rezidiv, Langzeit-EKG, Kathetergeführte Ablation, Kryoballon, Radiofrequenzablation

## Abstract

**Background:**

This retrospective observational study investigated the relationship between heart rate variability (HRV) and atrial fibrillation (AF) recurrence after pulmonary vein isolation (PVI) by cryoballoon or radiofrequency ablation (RF).

**Methods:**

We enrolled 497 patients who underwent PVI using first-generation cryoballoon (CB1), second-generation cryoballoon (CB2), or RF. We analyzed HRV as a surrogate for modulation of the intrinsic autonomic nervous system using 24‑h Holter recordings 1 or 2 days after the procedure and compared the recurrence and non-recurrence group with regard to ablation methods. Furthermore, we calculated recurrence-free survival (RFS) below/over HRV cut-off values for the whole study population and separately for each ablation method.

**Results:**

All except one of the five time-based HRV parameters analyzed were significantly lower in the non-recurrence group than in the recurrence group after CB2. Only a trend toward lower HRV for the non-recurrence group was found after RF and no remarkable differences were detected after CB1. The HRV parameters below their calculated cut-off were associated with a significantly higher RFS rate 2 years after CB2. This also applied to root mean sum of squared distance (rMSSD) and the percentage of adjacent NN interval differences greater than 50 ms (pNN50) after RF. No differences were found regarding CB1. Concerning rMSSD, the sensitivity, specificity, and difference in RFS increased when using cut-offs that were calculated including only CB2 patients. Multivariate cox regression analysis showed that low rMSSD values could independently predict AF recurrence after adjusting for covariates (hazard ratio: 0.50; *p* < 0.001).

**Conclusion:**

Low values of rMSSD early after a PVI could independently predict AF recurrence, especially after CB2.

**Supplementary Information:**

The online version of this article (10.1007/s00059-023-05201-6) contains supplementary material, which is available to authorized users.

## Abstract (de)

**Hintergrund:**

In der vorliegenden retrospektiven Beobachtungsstudie wurde der Zusammenhang zwischen der Herzfrequenzvariabilität (HRV) und Vorhofflimmer-Rezidiven (VHF) nach Pulmonalvenenisolation (PVI) mittels Kryoballon oder Radiofrequenzablation (RF) untersucht.

**Methoden:**

In die Studie wurden 497 Patienten eingeschlossen, bei denen eine PVI mit einem Kryoballon der ersten Generation (CB1), einem Kryoballon der zweiten Generation (CB2) oder RF erfolgte. Dabei wurde die HRV als Surrogat für die Modulation des intrinsischen autonomen Nervensystems anhand eines 24-h-Langzeit-EKG einen oder 2 Tage nach dem Eingriff untersucht und zwischen Rezidiv- und Nicht-Rezidivgruppe in Bezug auf die Ablationsmethode verglichen. Außerdem wurde das rezidivfreie Überleben („recurrence-free survival“, RFS) unter/über den HRV-Grenzwerten für die gesamte Studienpopulation und separat für jede Ablationsmethode ermittelt.

**Ergebnisse:**

Bis auf einen waren nach CB2-Behandlung alle der 5 untersuchten zeitabhängigen HRV-Parameter in der Gruppe ohne Rezidiv signifikant niedriger als in der Gruppe mit Rezidiv. Nur eine Tendenz in Richtung einer niedrigeren HRV wurde für die Gruppe ohne Rezidiv nach RF festgestellt, und nach CB1-Behandlung bestanden keine nennenswerten Unterschiede. Die HRV-Parameter unter dem jeweiligen berechneten Grenzwert waren mit einer signifikant höheren RFS-Rate 2 Jahre nach CB2-Behandlung verbunden. Das galt auch für den mittleren quadratischen Abstand („root mean sum of squared distance“, rMSSD) und den Anteil nebeneinanderliegender Normal-zu-normal(NN)-Intervalldifferenzen > 50 ms (pNN50) nach RF. Nach CB1 waren keine Unterschiede feststellbar. Sensitivität, Spezifität und Unterschied beim RFS von rMSSD nahmen zu, wenn Grenzwerte angewandt wurden, die nur unter Einschluss von CB2-Patienten berechnet wurden. Die multivariate Cox-Regressionsanalyse zeigte, dass nach Adjustierung für Kovariablen niedrige rMSSD-Werte unabhängig ein Rezidiv eines VHF nach vorhersagen konnten (Hazard Ratio: 0,50; *p* < 0,001).

**Schlussfolgerung:**

Niedrige rMSSD-Werte, die früh nach einer PVI auftraten, konnten in unabhängiger Weise ein VHF-Rezidiv vorhersagen, insbesondere nach CB2-Behandlung.

## What’s new?

- Low rMSSD values 1–2 days after cryoballoon or radiofrequency ablation can independently predict atrial fibrillation (AF) recurrence
- rMSSD is better suited for predicting AF recurrence after second-generation cryoballoon ablation than after radiofrequency ablation
- rMSSD was not able to predict AF recurrence after the outdated first-generation cryoballoon ablation

Atrial fibrillation (AF) is the most common permanent cardiac arrhythmia and a further rise in prevalence is still expected for Western nations [1]. Since the disease is associated with a significant burden of morbidity and mortality as well as financial strain, early detection and effective treatment of the arrhythmia remains an important goal. Regarding treatment, progress has been made using either cryo- or radiofrequency ablation for catheter-guided pulmonal vein isolation (PVI), which represents an effective tool to achieve rhythm control and, according to recent guidelines, should be considered as first-line therapy for a specific group of patients [1]. However, even for this patient group, a significant proportion of patients suffer from recurrence, with a single-procedure success rate between 66.6% for paroxysmal and 51.9% for non-paroxysmal AF 1 year after ablation [2], showing the need for additional therapeutic approaches. Concerning ablation success, a variety of predictors have been discussed, ranging from certain comorbidities to general patient characteristics such as age or sex. A decreased heart rate variability (HRV) is among these predictors; it can be obtained relatively easily through 24‑h Holter or (ultra-)short-term recordings and represents autonomous nervous system (ANS) activity. Since the origin and maintenance of AF are partly attributed to a disbalance between the sympathetic and parasympathetic nervous system [3] and modulation of the intrinsic ganglionated plexi as part of the ANS in addition to PVI has been shown to increase ablation success rate [4], HRV could conceivably be a useful proxy for predicting ablation success.

We therefore evaluated the relation between HRV and AF recurrence in patients after PVI using first-generation cryoballoon (CB1), second-generation cryoballoon (CB2), or radiofrequency ablation (RF).

## Methods

### Study population

In total, 497 patients were included in this retrospective observational study (for patient characteristics, see Table 1). Initially, we gathered data from 1522 patients who underwent either CB1, CB2, or RF from January 2011 to August 2017 in our center. CB2 was introduced in late April 2014 and completely superseded CB1 from that point on. We excluded patients with repeated ablation (*n* = 378), who had no recordings or unsuitable Holter recordings (*n* = 588), who had less than 15 h recording time (*n* = 5), and those who had missing follow-up data (*n* = 54). The follow-up period was limited to 24 months.

**Table 1:** Study population characteristics

		CB1	CB2	RF	*p*
Number		129	172	196	–
Age		58.4 ± 12.5	64.8 ± 10.0	60.7 ± 12.8	< 0.001
Gender	Male (%)	59 (46)	99 (58)	92 (47)	0.061
	Female (%)	70 (54)	73 (42)	104 (53)	–
BMI		28.7 ± 4.3	29.4 ± 4.9	28.0 ± 4.4	0.043
AF type	Paroxysmal (%)	118 (92)	135 (79)	127 (65)	< 0.001
	Persisting (%)	11 (8)	37 (21)	69 (35)	–
Smoking state	Non-smoker (%)	118 (91)	157 (91)	189 (96)	0.008
	Smoker (%)	10 (8)	6 (4)	4 (2)	–
	Ex-smoker (%)	1 (1)	9 (5)	3 (2)	–
CHF NYHA	No CHF (%)	118 (91)	147 (85)	177 (90)	0.536
	I (%)	4 (3)	5 (3)	3 (2)	–
	II (%)	6 (5)	15 (9)	12 (6)	–
	III (%)	1 (1)	5 (3)	4 (2)	–
	IV (%)	0 (0)	0 (0)	0 (0)	–
CAD (%)		13 (10)	45 (26)	22 (11)	< 0.001
Status post AMI (%)		2 (2)	7 (4)	3 (2)	0.217
Diabetes (%)		14 (11)	29 (17)	19 (10)	0.094
Hypertension (%)		84 (65)	136 (79)	121 (62)	0.001
Status post stroke (%)		3 (2)	8 (5)	7 (4)	0.564
Amiodaron (%)		38 (30)	42 (24)	55 (28)	0.583
Flecainide (%)		47 (36)	30 (17)	40 (20)	< 0.001
Flecainide PIP (%)		0 (0)	2 (1)	7 (4)	0.045
β‑Blocker (%)		112 (87)	144 (84)	177 (90)	0.169
CHA_2_DS_2_-VASc Score		1.8 ± 1.4	2.6 ± 1.5	2.0 ± 1.5	0.007
Mean timepoint of latest follow-up		31.4 ± 26	22.8 ± 13.7	31.7 ± 22	< 0.001
Recurrence-free rate after 2 years		57.3%	69.2%	72.2%	0.032

Continuous variables are shown as the mean ± SD and categorical variables as the number (%)

*AF* atrial fibrillation, *BMI* body mass index, *CHF* congestive heart failure, *CAD* coronary artery disease, *AMI* acute myocardial infarction, *PIP* pill-in-the-pocket, *CB1* first-generation cryoballoon, *CB2* second-generation cryoballoon, *RF* radiofrequency ablation, *SD* standard deviation

### Ablation procedure

The decision of whether to schedule patients for cryoballoon ablation or RF was made according to clinical judgment with regard to patient characteristics and the preference of the examiner.

The Cryoballoon (CB) and RF procedure and their preparation concerning imaging and anticoagulation have been previously described in detail [5].

In brief, the CB or RF catheter were delivered via transseptal puncture.

For CB, using the medtronic arctic front (CB1) or medtronic arctic front advance (CB2), each pulmonary vein was isolated (confirmed via entrance or exit block using Achieve, Medtronic spiral mapping catheter) after verifying occlusion.

For RF, we performed encircling PVI using the Thermocool (Biosense Webster) irrigated tip catheter and utilized a Lasso-catheter (Biosense Webster) to confirm isolation of the pulmonary veins.

### 24-h Holter recordings and HRV

All patients underwent at least 15-h-long Holter recordings (AMEDTEC EP8-S) 1–2 days after the procedure. The HRV parameters were analyzed using AMEDTEC ECGpro. The five following time-domain HRV parameters were analyzed: standard deviation of normal-to-normal (NN) intervals (SDNN; ms), standard deviation of the average NN intervals for each 5‑min segment (SDANN; ms), mean of the standard deviations of all the NN intervals for each 5‑min segment (SDNN-Index; ms), root mean square of the differences between adjacent NN intervals (rMSSD; ms); and the percentage of adjacent NN interval differences greater than 50 ms (pNN50; %).

### Patient follow-up

After ablation and discharge from our hospital, medical attendance of our patients was taken over by outpatient care, which scheduled follow-up visits 3–6 months after the ablation and then yearly after the first follow-up visit (thus 15–18 months after the ablation in the observation period for our study). Additionally, patients were encouraged to call in for an unscheduled visit if they were to experience recurrence-related symptoms. Recurrence was defined as ECG-recorded patterns typical for AF lasting at least 30 s and occurring at least 3 months after the ablation.

### Statistical analysis

All continuous variables are expressed as mean values (± standard deviation). Differences in baseline characteristics between the different ablation methods were compared using the Kruskal–Wallis test due to missing normal distribution, whereas differences in results were compared using the *t* test or Welch test if there was a difference in variances. Categorical variables are expressed as numbers and percentages and were compared using the chi-squared test. To test for homogeneity of variance, we used Levene’s test. Recurrence-free survival was analyzed using the Kaplan–Meier estimator with a log-rank test and adjustment for other possible risk factors was performed using Cox regression analysis. Furthermore, we used receiver operating characteristics (ROC) to determine cut-off values for our HRV parameters to conduct survival analysis. Statistical significance was defined as a two-sided *p* value smaller than 0.05. The software used to manage our database and to conduct statistical analysis was SPSS 26.0.0.0.

## Results

### Population characteristics and ablation outcome

Of the 497 patients included in our study, 129 had been referred to CB1, 172 to CB2, and 196 to RF. The mean timepoint of the latest follow-up overall was 28.5 month and the median 23.5 months. In the whole population, freedom of recurrence after 2 years was 70.4%. For the baseline characteristics of each group, see Table 1. The patients were distributed equally regarding gender, status post stroke, and status post acute myocardial infarction, diabetes, and the intake of amiodaron or beta-blockers. Statistically significant differences were found for age, body mass index, AF type, smoking rates, coronary heart disease, hypertension, use of class IC antiarrhythmics, CHA_2_DS_2_-VASc Score, and mean timepoint of latest follow-up. The freedom of recurrence rate 2 years after CB2 tended toward improved recurrence-free survival compared to CB, falling just short of reaching significance (*p* = 0,057).

### HRV differences between recurrence group and non-recurrence group according to ablation method

**Fig. 1:**
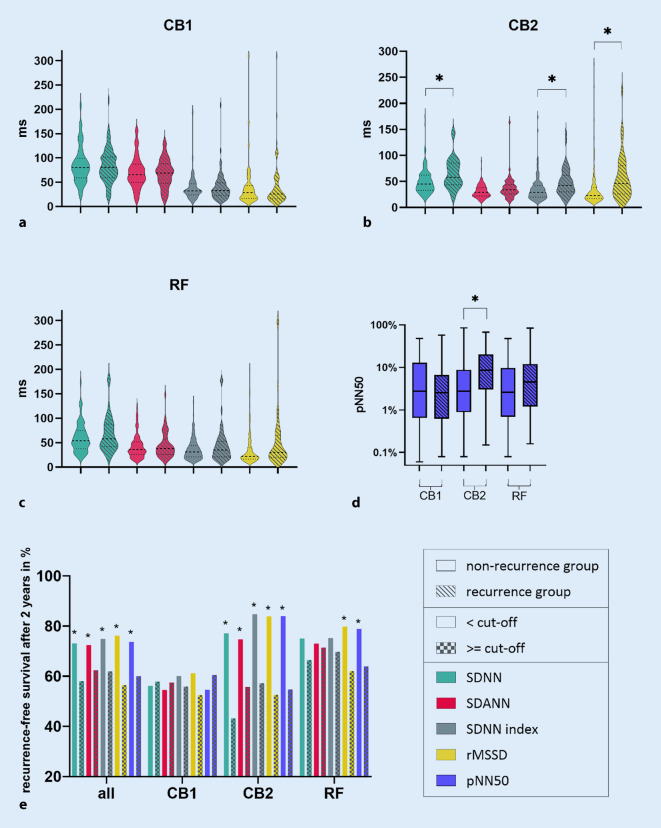
**a**–**d** Differences in heart rate variability (HRV) parameters comparing recurrence and non-recurrence group for each ablation method; *p < 0.02. **a** For CB1; **b** for CB2; **c** for RF; **d** regarding pNN50 for CB1, CB2 and RF; **e** comparison of recurrence-free survival (*RFS*) after 2 years below and over the respective cut-off of HRV parameters (SDNN: 69.5 ms; SDANN: 38.5 ms; SDNN index: 29.5 ms; rMSSD: 28.5 ms, pNN50: 3.845%) broken down for the general study population and each ablation method; *p < 0.02 comparing the group below and over the respective cut-off. *CB1* first-generation cryoballoon, *CB2* second-generation cryoballoon, *RF* radiofrequency ablation

In order to gain insight into potential discriminatory features of HRV parameters between the recurrence and non-recurrence group, we first visualized the data using violin plots (Fig. 1a–c) or a box plot for pNN50 (Fig. 1d). Comparing every individual HRV parameter between the two groups and per ablation method, we found that for CB2 all of them were significantly lower in the non-recurrence group, the exception being SDANN (*p* = 0.061). The biggest difference was found for rMSSD. No significant differences were found for RF and CB1.

### HRV cut-offs and recurrence-free survival for each ablation method

Based on these promising data, we next aimed to identify HRV cut-off values able to differentiate the recurrence from non-recurrence group after 2 years. Using the whole study population as starting point, we performed ROC analysis to determine cut-off values for every HRV parameter. Figure 1e gives an overview of the determined cut-offs (SDNN: 69.5 ms; SDANN: 38.5 ms; SDNN index: 29.5 ms; rMSSD: 28.5 ms, pNN50: 3.845%) as well as the percentage of patients above and below the cut-off with a recurrence-free survival of 2 years. This is shown for the whole study population and for each ablation method (for exact numbers, see Table 8 in supplementary data). Regarding CB2 and the general study population, our data show significantly higher recurrence-free survival rates in the group below the cut-off. The differences between recurrence-free survival above and below the cut-offs are visibly higher for CB2 (e.g., rMSSD 31.3% for CB2 vs. 19.7% for whole study population). For RF, recurrence-free survival is consistently higher for patients below the cut-off, that difference being significant in rMSSD (17.7%) and pNN50 (15%). For CB1, however, there are only small, insignificant differences in recurrence-free survival, sometimes favoring the one, sometimes favoring the other group.

### Cox regression for the most applicable predictor of AF recurrence

Because the chosen HRV parameters are almost completely highly correlated among themselves (see Table 9 in supplementary data as well as [6]) we decided to determine the most applicable parameter for predicting AF recurrence. For this purpose, we conducted a multivariate Cox regression including all five of parameters. In this regression model, the rMSSD cut-off was the only independent predictor for AF recurrence, cutting the risk for AF recurrence for patients below the cut-off in half (HR = 0.50; 95% CI: 0.29–0.85; *p* = 0.01). With rMSSD also featuring the highest area under the ROC curve (AUC), the highest Youden’s index, and the largest effect in the Kaplan–Meier analysis of the whole study population, we found rMSSD to be the most applicable predictor for AF recurrence in our study population.

### Sensitivity and specificity of RMSSD cut-off for the general study population and CB2

**Fig. 2:**
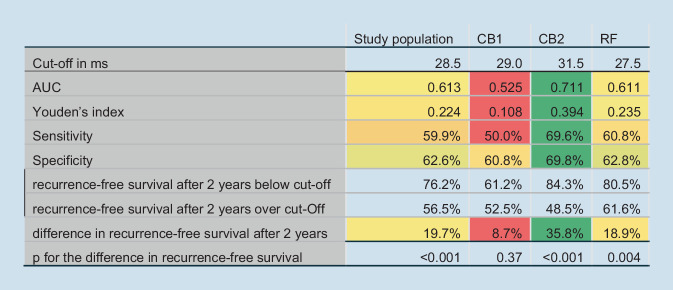
Results of receiver operating characteristics (ROC) analysis for root mean sum of squared distance conducted for the general study, color-coded for better overview: Lower values are *red*, higher values *green* and *yellow* values in between. population and for every ablation group. *AUC* area under the ROC curve, *CB1* first-generation cryoballoon, *CB2* second-generation cryoballoon, *RF* radiofrequency ablation

After determining rMSSD as the most applicable predictor, the results reported in Fig 1e suggest that CB2 might be the ablation method most suitable for using an HRV parameter as a predictor. While the previously reported cut-off values were calculated using the whole study population, we wanted to compare sensitivity, specificity, and recurrence-free survival between our study population and those patients having undergone CB2. For this purpose, we conducted one separate ROC analysis for each ablation method and determined different cut-offs, which we used to determine recurrence-free survival. The results are summarized in Fig. 2. The difference in recurrence-free survival below and above the cut-off increased by 16.1% in CB2 compared to the study population. The AUC increased by 0.1, Youden’s index by 0.17, sensitivity by 9.7%, and specificity by 7.2%.

### Adjusting for other risk factors

Using multivariate Cox regression, we adjusted for other previously reported risk factors. The rMSSD cut-off continued to be an independent predictor for AF recurrence but suffering from heart failure (NYHA I–IV) was also independently associated with a higher risk for AF recurrence. On the contrary, having undergone RF or suffering from coronary heart disease or hypertension was independently associated with a lower risk for AF recurrence, the same trend also applying to patients having undergone CB2. The results are summarized in Table 2.

**Table 2:** Results of multivariate Cox regression analysis adjusting for other previously reported risk factors for recurrence

	Significancy	Hazard ratio	95% CI for hazard ratio	
			Lower	Upper
Age	0.08	1.014	0.998	1.03
BMI	0.818	0.996	0.958	1.034
rMSSD cut-off (below:above)	*<* *0.001*	0.495	0.35	0.699
Gender (male:female)	0.159	1.274	0.91	1.738
AF type (paroxysmal:pers.)	0.296	1.25	0.823	1.898
Heart failure (yes:no)	*<* *0.001*	2.531	1.586	4.039
CAD (yes:no)	*0.014*	0.512	0.3	0.875
Hypertension (yes:no)	*0.048*	0.683	0.469	0.997
β‑Blockage (yes:no)	0.088	1.64	0.928	2.898
CB1 (reference)	0.058	–	–	–
RF	*0.021*	0.619	0.411	0.93
CB2	0.098	0.7	0.458	1.069

*CI* confidence interval, *BMI* body mass index, *AF* atrial fibrillation *CAD* coronary artery disease, *CB1* first-generation cryoballoon, *CB2* second-generation cryoballoon, *RF* radiofrequency ablation

## Discussion

Catheter-based ablation methods are a powerful tool to achieve long-term rhythm control when treating AF. However, not all patients seem to benefit equally from the procedure, as approximately one third of them suffer from recurrences within 1 year. To date, there is still a lack of viable characteristics to identify this group, which could benefit from a different follow-up-treatment. With our study, we demonstrate that low rMSSD values a few days after the ablation can independently predict AF recurrence. This relationship is strongest for CB2 and therefore better suited to predict recurrence after CB2 than after RF. When regarding only the mostly superseded CB1, this association is the weakest and insignificant.

As reported by Pandya et al. [7], in our data too recurrence-free survival was improved by CB2 (*p* = 0.057) compared to CB1, falling just short of significance. Likewise, as stated by the most recent guidelines [1], we also found CB2 to be noninferior to RF regarding efficiency, thus confirming the validity of our dataset.

Before we identified rMSSD as a predictor for recurrence, we first examined the differences in mean HRV between the recurrence and non-recurrence group, finding that suffering from AF recurrence is associated with significantly higher HRV after CB2 (except for SDANN). This led us to attempt to identify specific cut-offs for differentiating between groups with either high or low risk for recurrence, which we found for all HRV parameters. Thus, our next step was to compare them and find the most applicable: rMSSD, as confirmed by the first Cox regression and validated as an independent predictor by the second analysis. Our earlier results also suggested that the calculated cut-off for rMSSD might show different predictive capabilities depending on the ablation method. Therefore, instead of only using the whole population as basis for our cut-off calculation, we recalculated the best cut-off for every ablation method, confirming our hypothesis that rMSSD is best suited to evaluate recurrence risk after CB2.

Several studies in the past have demonstrated a relation between low HRV after ablation and lower recurrence risk. However, the HRV parameters that have been found to be predictive for AF recurrence differ to some extent between those studies. Wang et al. reported significantly reduced SDNN and rMSSD for the non-recurrence group, but unlike us determined SDNN as the sole independent predictor [8]. Two other studies identified only frequency-domain HRV parameters as independent predictors for recurrence [9, 10] and Zhu et al. found rMSSD, pNN50, and high-frequency components (HF) as independent predictors for AF recurrence [11]. These three studies, however, only included patients who received RF, which could explain some of the differences compared to our study given we included also the first two generations of cryoballoon ablation. We found only one study including patients treated with CB2, which observed significantly reduced rMSSD, HF, and low-frequency components after ablation only in the non-recurrence group but measured not until 3 months after the procedure [12]. Oswald et al. did not find a relation between AF recurrence and attenuated HRV but they used only CB1, and agreed with our estimation that HRV is not suited to predict recurrence for this superseded method [13]. To the best of our knowledge, this is the first study to examine the relationship between HRV and AF recurrence for more than 300 patients while at the same time including patients after CB1, CB2, and RF. Thus, while comparing well with the aforementioned studies in confirming the existence of said relationship with a larger sample, we also add novelty regarding the notion that the strength of this relation might depend on the ablation method.

Taking into consideration that rMSSD “is the primary time-domain measure used to estimate vagally-mediated changes reflected in HRV” [14], our finding of rMSSD as the sole independent predictor for recurrence is consistent with the hypothesized vagal withdrawal happening during ablation. Pappone et al. were among the first to report a link between HRV attenuation and AF recurrence. They mapped parasympathetic innervation around the pulmonary veins and proposed the addition of complete vagal denervation to PVI after they found it to significantly reduce AF recurrence [15], thus identifying a new potential target site for ablation. The intrinsic part of the extensive autonomous innervation of the heart is made of ganglionated plexi (GP), which are located mainly in fat pads around the pulmonary vein ostia [4]. While it has been shown that adjunctive ablation of these GP can increase the success rate of pulmonary vein ablation [4], this technique is not well established. It has been demonstrated that PVI alone attenuates HRV [12, 13] and a more recent study observed vagal responses and HRV attenuation in a similar fashion for both PVI and PVI with GP ablation [16], suggesting that PVI alone might produce GP damage to at least some degree. With the knowledge that the origin and maintenance of AF are partly attributed to a disbalance in the ANS [3], this could explain why HRV could be a useful predictor for AF recurrence.

Concerning our conclusion that HRV is better suited to predict recurrence after CB2 than after RF, we hypothesized that this could be due to the circumstance that ablation via cryoballoon increases myocardium-specific enzymes more strongly after the ablation and causes wider lesions than RF [17], suggesting that CB2 creates more extensive lesions and thus modulates/destroys more GP. The same mechanism could explain why for CB1, HRV is not as applicable for predicting AF recurrence: only four vs. six cryosource emission holes as used with CB2 result in a smaller cooling surface area [18] and potentially in fewer destroyed GP.

Apart from confirming rMSSD as an independent predictor for AF recurrence, our Cox regression also suggests that suffering from heart failure increases the risk for recurrence, which is mostly consistent with previous findings [19]. However, suffering from coronary disease or hypertension seems to be a protective factor in our study, which stands in contrast to previous findings [20]. There are multiple possible explanations for this discrepancy: Firstly, Hiraya et al. reported a lower risk of recurrence for patients with coronary disease who underwent percutaneous coronary intervention [20] and we did not document how many of our patients with coronary disease might have profited from that measure. Secondly, since our co-occurrence of coronary disease and heart failure (see Tables 4 and 5 in supplementary data) is far lower than described in the literature [21] and our study was conducted retrospectively, it may be that one or both comorbidities were poorly documented. The third approach to an explanation also applies to hypertension being a protective factor, because it suggests that patients suffering from one of both conditions might profit from a more extensive antiarrhythmic drug treatment. The percentage of patients with coronary disease/hypertension receiving beta-blockers is significantly higher than the percentage of patients with beta-blockers without those conditions (see Tables 6 and 7 in supplementary data). However, our multivariate Cox-regression analysis could not detect a significantly decreased risk for recurrence for patients taking beta-blockers and on the contrary the trend pointed toward an increased risk for this group. A future prospective study would not have to rely on proper documentation in the past and could thus circumvent part of these issues.

### Limitations

Our study was conducted retrospectively in a single center and the baseline characteristics were not all similar among the three different ablation methods. Since we only obtained HRV data shortly after the ablation, it is not safe to say that the difference between the recurrence and non-recurrence group appeared only after the procedure and did not already exist before it. To prevent patients from being wrongly categorized into a high-/low-risk group, it would have been more accurate to measure the difference in HRV before and after the ablation. Furthermore, our study might have experienced selection bias since we excluded patients whose Holter recordings were not suitable for HRV analysis, thus possibly ignoring a large fraction of patients with early recurrence.

## Conclusion

In conclusion, a decrease in the root mean square of the differences between adjacent NN intervals is an independent predictor for atrial fibrillation recurrence and could be a feasible first step, especially after second-generation cryoballoon procedures, to categorize patients into a high- or low-risk-group in order to facilitate decisions concerning follow-up treatment. Future research is needed.

### Supplementary Information

§§contingency tables, HRV correlation table and table with numeric values for figure 1e

## Declarations

This research study was conducted retrospectively from data obtained for clinical purposes, after consultation with the institutional ethics committee and in accordance with national legal requirements. As far as an informed consent into a retrospective analysis of patient data is not feasible, patient data collection and management were approved by the local medical ethics committee.
