# Supplementary material for: Heart rate variability as a predictor of successful catheter-guided pulmonary vein isolation for atrial fibrillation
Source: Herz. 2023 Aug 17;49(2):147–54. doi: 10.1007/s00059-023-05201-6 (PMC10917838; doi:10.1007/s00059-023-05201-6)
Supplement: Supplementary file 1 — §§contingency tables, HRV correlation table and table with numeric values for figure 1e [file 59_2023_5201_MOESM1_ESM.docx]

## Supplementary data

**Table 4: contingency table for the coincidence of congestive heart failure (CHF) and presence of coronary artery disease (CAD)**

|  | |  | No CHF | CHF | total |
| --- | --- | --- | --- | --- | --- |
|  | no CAD |  | 380 | 37 | 417 |
|  | CAD |  | 62 | 18 | 80 |
| total | |  | 442 | 55 | 497 |

**Table 5: comparing the presence of both coronary artery (CAD) and congestive heart failure (CHF) between our and other study populations**

|  | |  | our study population | | Gheorgida et al.(43) | Morbach et al.(42) | |
| --- | --- | --- | --- | --- | --- | --- | --- |
|  | n | |  | 497 | 43 568 | 509 |  |
|  | % of CAD patients with CHF | |  | 32.7 | - | 96.1 |  |
|  | % of CHF patients with CAD | |  | 22.5 | 62.0 | - |  |

**Table 6: contingency table for the coincidence of ß-blocker-usage and presence of coronary artery disease (CAD)**

|  | | no CAD | CAD | total | p-value |
| --- | --- | --- | --- | --- | --- |
| ß-blocker | no | 60 | 4 | 64 |  |
|  | yes | 357 | 76 | 433 |  |
| Total | | 417 | 80 | 497 | 0,022 |

**Table 7: contingency table for the coincidence of ß-blocker-usage and presence of hypertonia**

|  | | no hypertonia | hypertonia | total | p-value |
| --- | --- | --- | --- | --- | --- |
| ß-blocker | no | 37 | 27 | 64 |  |
|  | yes | 119 | 314 | 433 |  |
| total | | 156 | 341 | 497 | <0,001 |

**Table 8: recurrence-free survival above and below our respective HRV cut-offs shown for the whole study population and each ablation group, * p<0,02**

|  |  | whole study population | CB1 | CB2 | RF |
| --- | --- | --- | --- | --- | --- |
|  |  | recurrence-free survival | recurrence-free survival | recurrence-free survival | recurrence-free survival |
| SDNN | <69.5 | 73.1 % * | 56.2 % | 77.1 % * | 75.1 % |
|  | >=69.5 | 58.1 % | 57.9 % | 43.2 % | 66.5 % |
| SDANN | <38.5 | 72.4 % * | 54.5 % | 74.7 % * | 73 % |
|  | >=38.5 | 62.4 % | 57.5 % | 55.7 % | 71.4 % |
| SDNN index | <29.5 | 74.9 % * | 60.1 % | 84.7 % * | 75.2 % |
|  | >=29.5 | 61.9 % | 55.9 % | 57.2 % | 69.8 % |
| rMSSD | <28.5 | 76.2 % * | 61.2 % | 83.9 % * | 79.8 % * |
|  | >=28.5 | 56.5 % | 52.5 % | 52.6 % | 62.1 % |
| pNN50 | <3.845 | 73.7 % * | 54.6 % | 84 % * | 78.9 %* |
|  | >=3.845 | 60 % | 60.5 % | 54.7 % | 63.9 % |

**Table 9: correlation according to Pearsson of different HRV parameters among themselves, * p≤0,005**

|  | | SDANN | SDNN index | rMSSD | pNN50 |
| --- | --- | --- | --- | --- | --- |
| SDNN |  | 0.779^*^ | 0.790^*^ | 0.672^*^ | 0.562^*^ |
| SDANN |  |  | 0.259^*^ | 0.127^*^ | 0.060 |
| SDNN index |  |  |  | 0.917^*^ | 0.827^*^ |
| rMSSD |  |  |  |  | 0.874^*^ |
